# Electrosurgical bipolar vessel sealing versus conventional clamping and suturing for total abdominal hysterectomy

**DOI:** 10.12669/pjms.38.1.4197

**Published:** 2022

**Authors:** Mustafa Ulubay, Mehmet Ferdi Kinci, Ramazan Erda Pay, Murat Dede

**Affiliations:** 1 Mustafa Ulubay, Assistant Professor, University of Health Sciences, Gulhane Education and Research Hospital, Obstetrics and Gynecology Department, Ankara, Turkey; 2 Mehmet Ferdi KINCI, M.D, Mugla Sitki Kocman University Education and Research Hospital, Obstetrics and Gynecology Department, Mugla, Turkey; 3 Ramazan Erda Pay, M.D, Bingol State Hospital, Obstetrics and Gynecology Department, Bingöl, Turkey; 4 Prof. Murat DEDE, Special Anadolu Health Center, Obstetrics and Gynecology Department, Istanbul, Turkey

**Keywords:** Conventional Sutures, Operation time, Total Abdominal Hysterectomy, Vessel Sealing System

## Abstract

**Objectives:**

To compare the use of Electrosurgical bipolar vessel sealing LigaSure™ small jaw instrument (LSJI) with conventional suture ligation in total abdominal hysterectomy (TAH).

**Methods:**

In this retrospective study 80 patients who underwent hysterectomy in the Gynecology and Obstetrics Department of Gulhane Education and Research Hospital between April 2017 and August 2018 were included. Two different groups that underwent Electrosurgical bipolar vessel sealing LigaSure™ small jaw instrument (LSJI) and conventional suture ligation in hysterectomy operation were analyzed retrospectively. The parameters evaluated and compared between the two groups include operation time, intraoperative blood loss, duration of hospitalization and incision length.

**Results:**

Among the parameters we compared between the two groups, there was no statistically significant difference between the amount of intraoperative blood loss (p:0.68) and the incision length (p:0.65). Among the parameters we compared between the two groups, a statistically significant difference was observed between the operation time (p:0.016) and the duration of hospitalization (p:0.01).

**Conclusion:**

Our comparison of LSJI vs. conventional ligation in hysterectomy revealed a significant difference only in operative time, where surgeries involving conventional ligation were shorter. On the other hand, incision length was evaluated in our study which has not been addressed in previous studies. There is also a need for multi-center studies that include more patients and evaluate cost-effectiveness.

## INTRODUCTION

Hysterectomy is one of the most commonly performed surgical procedures in women. Estimates suggest that one in nine women will undergo hysterectomy during their lifetime and that approximately 600,000 procedures are performed each year in the United States.^1^ The first technique introduced was total abdominal hysterectomy (TAH) which was mostly succeeded by minimally invasive methods over time. Although vaginal hysterectomy (VH) remains the gold standard, technological advances have enabled a trend towards Laparoscopic Hysterectomy (LH) and Robotic Hysterectomy (RH), numerous variations of which have also been described.^2^-^4^ Lately, conventional suture ligation techniques employed in such procedures are being replaced by laser applications and various electrothermal and ultrasonic coagulation methods.^5^,^6^

During electrothermal coagulation, a controlled high-power current at low voltage from a device is used to melt the collagen and elastin in tissue, leading to permanent fusion of the vascular layers and obliteration of the lumen. The device fuses vessels up to 2–7 mm in diameter.^7^ In addition to minimally invasive procedures, the use of electrothermal coagulation has been expanded within years to hemorrhoidectomy, thyroidectomy, and certain abdominal surgeries.^5^,^6^ Conventional versus technology-aided hysterectomy modalities have been compared by some studies both for intraoperative and postoperative parameters.

In the present study, we have evaluated blood loss, size of incision, duration of operation, volume of the uterus, and length of hospitalization with Ligasure vessel sealing system in comparison to conventional ligation at hysterectomy.

## METHODS

This retrospective study was approved by the local clinical research ethics committee (CREC Decision No: 2020-65). It was performed at the Gulhane Education and Research Hospital, Ankara, Turkey over the period from April 2017 to August 2018. Patient data were scanned through the hospital data system. The data of 63 TAH + Bilateral salpingo-oophorectomy (BSO) and 17 TAH + Bilateral salpingectomy patients who had the inclusion criteria were scanned.

### Pre-Surgical procedure:

All patients were questioned for their detailed medical history before their surgery. Their age, height, weight, body mass index (BMI), gravidity, parity, and history of previous abdominal surgery were recorded. During the course of their physical examination, bimanual and speculum examination was carried out followed by transvaginal ultrasound (TV US). Further imaging or diagnostic tests were requested when deemed necessary. Endometrial biopsy samples were collected from the patients at risk who presented with abnormal uterine bleeding. All patients had complete blood count and routine biochemical testing done before the surgery. Patients who experienced any concomitant surgical procedures and those who underwent hysterectomy for gynecologic cancer were excluded from this study.

### Surgical procedure:

For each case, perioperative prophylactic, intravenous (IV) first-generation cephalosporin antibiotic (cefazolin sodium 1 g) was administered. All patients underwent extrafascial TAH (Type-1) ± bilateral salpingo-oophorectomy or bilateral salpingectomy procedure. Abdominal access was gained through a transverse (Pfannenstiel) incision. Round ligament, suspensory ligament of the ovary or proper ovary ligament, uterine artery, cardinal ligament, and uterosacral ligaments were detached from the uterus. For conventional abdominal hysterectomy, clamping and cutting were followed by tying with polyglactin suture material (Vicryl size: 0, Ethicon, NJ, USA). In the LigaSure™ small jaw instrument (LSJI; Medtronic, Boulder, CO, USA) (Valleylab, CO, USA) method, clamping was followed by sealing and cutting. The closure of the vaginal cuff was secured a single-layer continuous running suture. Once hemostasis was checked, the peritoneum was closed with a 2/0 Vicryl suture™ (Polyglactin 910 Suture, Ethicon Co, USA) and fascia with a Vicryl suture size:0. In patients with a subcutaneous fat tissue thicker than 2 cm, a subcutaneous approximation suture was placed. The skin was then closed with a 4/0 Vicryl Rapide suture™ (Polyglactin 910 Suture, Ethicon Co, USA).

### Post-operative Follow-up:

Patients were given 75 mg I.M. Diclofenac Sodium BID (Diclomec 75 mg/3 mL IM Ampoule, Solution for Injection, Abdi Ibrahim, Istanbul, Turkey) used as postoperative analgesia. Study arms were compared in terms of their operative time, blood loss, postoperative complications, hospital stay, and incision length. In this comparison, the operative time was considered as the time elapsed from anesthesia induction to awakening. Postoperative hematocrit (HCT) values of the patients were measured at 8 h and 24 hour after the procedure. The length of incision was determined by a first-year resident using a ruler during the wound dressing applied at 24 hour after the procedure. Uterine volumes were calculated in cm^3^ by multiplying all three dimensions as reported in the pathology report.

### Statistical analysis:

Statistical analysis of data was performed using IBM SPSS (Statistical Package for Social Sciences) for Windows 15.0. Descriptive statistics (mean, standard deviation) were used to present the study data. Before quantitative characteristics were compared, the decision as to whether they have a normal distribution was made based on skewness and kurtosis of distribution. A comparison of groups was done using independent samples t-test in case of continuous variables and using the Chi-square test in case of categorical variables. For all results, the level of statistical significance was set to p<0.05.

### Evaluation of other studies about hysterectomy with vessel sealing systems:

Previous publications were identified through a search in “Google Scholar” and “Pubmed” without date restriction. The key words selected for the search were: hysterectomy, abdominal hysterectomy, vessel sealing system, LigaSure® Electrosurgical Vessel Sealer, and Conventional Suture Technique. We did not include unpublished papers. Our search produced 13 eligible hits. We have reviewed these papers to extract the study date, sample size (n), applied methods, amount of blood loss, duration of surgery, complication rates, and hospital stay (Table-I).

**Table I:** Review of previous studies.

Author(s)	Date	Device	Patient Number	Blood Loss	Operative Time	Complication	Hospital Stay
Maher M. *et al.*^20^	January 2009 - December 2009	Ligasure vessel sealing system	50 *vs.* 50	3.29 ± 2.02 *vs.* 3.90 ± 1.93 (p = 0.13) Hematocrit	59.98 ± 10.31 *vs.* 74.30 ± 12.81 min (p<0.0001)	Bladder injury, Vault bleeding, Wound infection 2% *vs.* Bladder injury 4%, Wound infection 2%	3.98 ± 0.62 *vs*. 4.04 ± 0.81 days; p = 0.68
Briones Landa CH. *et al.*^11^	March 2007 - February 2008	Bipolar plasmakinetics vessel sealing	47 *vs.* 47	209 ± 92 *vs.* 330 ± 113 mL (p<0.003),	82.9 ± 12.69 *vs*. 99.1 ± 18.4 min (p<0.001).	-	2.06 ± 0.24 *vs*. 3.2 ± 0.89 days, p<0.001
Wang K. et al.^10^	October 2013 - October 2015	Ligasure sealing vessel system	48 *vs.* 48 cervical cancer	473.28 ± 96.43 *vs.* 738.15 ± 102.81 mL p<0.05	161.79 ± 32.47 *vs.* 212.56 ± 31.05 min p<0.05	9 (18.8%) *vs.* 18 (37.5%) p<0.05	13.28 ± 3.62 *vs.* 16.97 ± 4.25 days p<0.05
Aydın C. et al.^9^	January 2010 - October 2010	Ligasure vessel sealing system	44 *vs.* 44 large uterus	0.99 ± 0.74 *vs.* 1.13 ± 0.81 HGB p = 0.328	109.91 ± 26.5 *vs.* 124.77 ± 35.51 min p = 0.029	Wound infection (2.2%) *vs.* Bladder injury, Hemorrhage (2.2%)	5.92 ± 2.63 *vs.* 5.95 ± 1.82 days
Rossetti D. et al.^12^	January 2001 – October 2013.	Ligasure vessel sealing system	23 *vs.* 26 peripartum hysterectomy	1900 (700-4000) *vs*. 2700 (800-8000) mL p = 0.001	110 (60-240) *vs.* 170 (85-320) min p = 0.06	6/23 (26%) *vs.* 4/26 (15%) p = 0.35	6 (4-9) *vs.* 8 (5-10) days p = 0.78
Türkçüoğlu I. *et al.*^8^	July 2010 - October 2010	Ligasure vessel sealing system	22 *vs.* 31	157.1 ± 89.1 *vs.* 142.3 ± 40.5 mL; p = 0.749	90.2 ± 20.6 *vs.* 92.1 ± 21.1 min; p = 0.962	-	3.6 ± 2.4 *vs*. 3.2 ± 1 days; p = 0.527
Hagen B. *et al.*^17^	June 2002 - April 2003	Ligasure vessel sealing system	16 *vs.*16	303 *vs.* 298 mL	61.7 *vs*. 54.5 min	Wound infection (18.75%), Wound rupture (6.25%) *vs.* Wound infection (6.25%), Vault bleeding (6.25%)	10 *vs.* 6 days
Bruno R. *et al.*^18^	-	Ligasure impact, using the Force Triad energy platform	-	80 *vs.* 122 mL (34% difference)	35 *vs*. 50 min (30% difference)	4 *vs.* 8 patients with blood transfusion	2.6 *vs.* 3.5 days
Lakeman M. *et al.*^19^	January 2005 – September 2006	Ligasure vessel sealing system	28 *vs.* 29	200 (33-1500) *vs*. 335 (70-1750) p = 0.08	69 (29-130) *vs.* 63 (38-124) min p = 0.62	Ileus requiring re operation, Fever of unknown origin, Thromboembolism 3% *vs.* Infected hematoma, Wound dehiscence, Pneumonia 3%	4 (2-32) *vs.* 5 (3-11) days; p = 0.26
Suprasongsin C. and Boonyakitanon M.^13^	November 2010 - December 2011	Electrosurgical Bipolar Vessel Sealing	30 *vs.* 30	248.33 ± 154.52 *vs*. 357.00 ± 245.34 mL; p = 0.04	70.03 ± 21.06 *vs.* 92.3 ± 26.54 min; p<0.001	Bladder injury, Infected wound, Infected vaginal stump 3.3% *vs.* Infected wound 6.6%, Infected vaginal stump 3.3%	4.13 ± 0.35 *vs.* 4.4 ± 0.67; p = 0.06
Dessole S. *et al.*^14^	August 2000	Bipolar electrocautery scissors	50 *vs.* 50	2.9 *vs.* 5.9 HTC%, p<0.001	91 ± 15 *vs.* 121 ± 32 min; p<0.01	There was no increase in the complication rate.	-
Lauroy A. *et al.*^15^	February 2005 - August 2018	Ligasure vessel sealing system	29 *vs.* 57 peripartum hysterectomy	3198 *vs.* 4223 mL; p = 0.02	45.62 (10-120) *vs.* 38.05 (8-114) min; p = 0.1	8 (27.5) *vs.* 19 (33.3); p = 0.25	-
Talaat A. and Makboul G. 16	March 2010 - January 2012	The Ultracision (Harmonic Shears) device	30 *vs.* 30	74.9 ± 56.7 *vs*. 139.4 ± 118.4 mL; p = 0.005	40.3 ± 19.7 *vs.* 55.6 ± 22.4 min; p = 0.003	10% *vs.* 10%	-

## RESULTS

Based on the discretion of the surgeon and the menopausal status of the women, 63 patients underwent TAH+BSO, and 17 patients underwent TAH+bilateral salpingectomy. Accordingly, in the TAH+BSO group, 30 patients were treated with LSJI and 33 patients were treated with the conventional method. In the TAH+bilateral salpingectomy group, on the other hand, 10 patients were treated with LSJI and 7 patients were treated with the conventional method. Patient indications for hysterectomy are given in Table-II.

**Table II:** Patients by indication for hysterectomy.

	LSJI n (%)	Conventional n (%)
Uterine Myoma	20 (50%)	19 (47.5%)
Endometriosis	5 (12.5%)	6 (15%)
Abnormal Uterine Bleeding	12 (30%)	12 (30%)
Endometrial Hyperplasia	3 (7.5%)	3 (7.5%)

The study arms had no significant difference in terms of gravidity, parity, BMI, or preoperative HCT values of patients (p>0.05) Table-III. In our postoperative analysis, HCT value at 24h after the procedure, uterine volume, incision length and duration of hospitalization were also not significantly different (p>0.05). On the other hand, operative time, HCT value at 8h after the procedure, and postoperative hospital stay were significantly different between the arms(p<0.05) Table-III and IV.

**Table III:** Demographic characteristics of the patients.

	LSJI (mean ± SD)	Conventional (mean ± SD)
Gravidity (n)	3.02±1.54	3.35±1.16
Parity (n)	2.1±0.84	2.67±0.97
BMI (kg/m^2^)	27.4±3.74	25.69±2.1

**Table IV:** Preoperative and postoperative characteristics of the patients.

	LSJI (mean ± SD)	Conventional (mean ± SD)	p-value
Preoperative HTC (%)	37.4±4.01	36.03±2.72	0.069
Postoperative 8 h HTC (%)	33.9±3.67	32.33±2.58	0.026
Postoperative 24 h HTC (%)	31.4±3.73	30.32±2.61	0.10
HTC reduction*	5.95±2.63	5.71±2.74	0,688
Uterine Volume (cm^3^)	472.5±412.8	392.51±128.04	0.24
Incision Length (cm)	15.5±1.9	14.81±1.27	0.65
Operative Time (min)	104.5±42.9	86.62±15.82	0.016
Duration of Hospitalization (days)	2.47±0.59	3.17±0.71	0.01

* HTC at postoperative 24 hour - preoperative HTC.

Postoperative complications experienced by the patients were wound infection in two patients and vaginal cuff hematoma in one patient at the LSJI arm, whereas one patient developed wound infection and one patient had cuff dehiscence in conventional ligation arm.

## DISCUSSION

Hysterectomy is the most common gynecological surgery across the globe. Although the advent of the minimally invasive techniques has led to a gradual decrease in the TAH rate, it remains the most frequent method.^2^ As the utilization of various technology-aids raised over the years, conventional techniques have been compared in numerous aspects versus these technological modalities (Table-IV). Here, we have compared the LSJI method and the conventional method of ligation applied in hysterectomy in terms of HTC decrease, uterine volume, length of incision, operative time, and hospital stay.

We have not detected a significant difference in HCT values as measured before the surgery and 24h after the surgery (p:0.069, p:0.10, respectively). On the other hand, HTC measured 8h after the surgery was significantly higher in the LSJI arm (p:0.026). Blood loss was estimated by subtracting the preoperative HTC value from the postoperative value at 24h, which did not yield a significant difference between the study arms (p:0.688). In the previous studies, blood loss has been estimated in mL depending on the change in either HCT or hemoglobin (HGB) or through EBL (estimated blood loss) calculation. Although some studies are suggesting that a bipolar vessel sealing system does not affect operative blood loss,^8^-^10^ there are also reports of a reduction in operative blood loss.^10^-^16^ Moreover, no p-value has been specified in some studies, which does not allow a make any robust inferences.^17^,^18^

Operative time is one of the parameters which is included among the criteria used in a comparison of LSJI vs. conventional methods in hysterectomy. In our cohort, operative time was longer in the LSJI arm as compared to the arm of the conventional method of ligation in hysterectomy (p:0.016). This result is in line with the results of Lauroy A et al. and Lakeman et al.^15^,^19^ In our opinion, the longer operative time in LSJI surgeries results from the uncontrolled minor bleedings and the need for additional sutures. In other studies, on contrary, a shorter duration of operation was counted when LSJI is employed than when conventional methods were used.^8^-^14^,^16^,^18^,^20^ Such studies argue LSJI is timesaving for surgery as it enables a single-step accomplishment of ligation which otherwise has to be done through clamping, cutting, and suturing.

Recently, cosmetic outcomes have a higher impact on the overall assessment of surgical success. In patients who are ineligible for minimally invasive surgical procedures, (laparoscopy/robotic surgery) location and size of the incision to conduct a laparotomy are of utmost importance. From the obstetrics point of view, the length of incision in Cesarean sections has been evaluated.^21^,^22^ The incision length, however, has not yet been explored in the hysterectomy setting. In our experience, hysterectomies implemented with LSJI vs. conventional ligation method were not statistically different in terms of incision length (p:0.65). A paramount effect on incision length is exerted by the size of the uterus to be removed. In our study, the mean uterine volumes were not significantly different between the groups (p:0.24) which allows for a healthier evaluation of incision length.

Duration of hospitalization due to a surgical procedure is crucial to avoid hospital infections and to improve cost-effectiveness. There was a significant difference in hospital stay between the two groups of our study (p:0.01). Although part of the previous studies has reported comparable results to ours,^6^,^12^,^19^,^20^ some studies achieved shorter inpatient stay for hysterectomy patients who were treated with LSJI.

### Limitations of the study:

The present study does not involve any post-operative pain or cost analysis. This design characteristic is one of the limitations of our study.

### Strengths of the study:

Incision length is evaluated in our study which has not been addressed in previous studies.

## CONCLUSION

Our comparison of LSJI vs. conventional ligation in hysterectomy revealed a significant difference only in operative time, where surgeries involving conventional ligation were shorter. In our study, the duration of hospital stays of patients who underwent surgery with LSJI was shorter. It is an advantage of operating with LSJI in protection against hospital infections, which increases as the duration of hospitalization increases. None of the other parameters included in our analyses showed any significant difference.

### Author’s Contribution:

**MU** conceptualized and design the study, reviewed the manuscript,

**MFK** wrote the manuscript,

**REP** collected data, made statistical analyzes,

**MD** perfomed surgical operations, revised the manuscript.
